# Research trends and hotspots of central sleep apnea: a bibliometric analysis

**DOI:** 10.3389/fneur.2025.1586667

**Published:** 2025-05-20

**Authors:** Yin-Chao Hao, Meng-Chu Zhu, Wei-Xuan Li, Jing-Jing Sha, Xue-Tong Dong, Song-Jun Wang, Chao-Long Lu

**Affiliations:** ^1^Department of Neurobiology, Hebei Medical University, Shijiazhuang, China; ^2^Functional Laboratory, Experimental Center for Teaching, Hebei Medical University, Shijiazhuang, China; ^3^Department of Laboratory Diagnostics, Hebei Medical University, Shijiazhuang, China; ^4^Undergraduate of College of Forensic Medicine, Hebei Medical University, Shijiazhuang, China; ^5^Hebei Key Laboratory of Forensic Medicine, Research Unit of Digestive Tract Microecosystem Pharmacology and Toxicology, Collaborative Innovation Center of Forensic Medical Molecular Identification, College of Forensic Medicine, Chinese Academy of Medical Sciences, Hebei Medical University, Shijiazhuang, China; ^6^Department of Medical and Pharmaceutical Informatics, Hebei Medical University, Shijiazhuang, China

**Keywords:** central sleep apnea, bibliometrics analysis, CiteSpace, VOSviewer, pathophysiology

## Abstract

Central sleep apnea (CSA), characterized by unstable ventilatory control during sleep, poses significant health risks, particularly in patients with cardiovascular comorbidities. This bibliometric analysis evaluated 1,687 CSA-related publications (2004–2025) from the Web of Science Core Collection. Annual publications surged post-2016, peaking in 2021 (115 articles), reflecting growing research interest. The U.S. and Germany dominated contributions, with American Journal of Respiratory and Critical Care Medicine as the top journal. Keyword analysis revealed three focal areas: CSA-heart failure interactions (e.g., mortality, ejection fraction), CSA mechanisms (e.g., hypercapnia, chemosensitivity), and clinical management (e.g., adaptive servo-ventilation, phrenic nerve stimulation). Emerging trends include pediatric CSA, pathophysiology, and AI-driven diagnostics. International collaboration and multidisciplinary approaches are critical for advancing CSA research. Limitations include database constraints and evolving literature. This study maps CSA research trends, highlights gaps, and guides future investigations into mechanisms, biomarkers, and personalized therapies.

## 1 Introduction

Central sleep apnea (CSA) is a subtype of sleep apnea characterized by a lack of respiratory power during sleep, resulting in hypo- or absent-ventilation and impaired gas exchange, and marked by unstable ventilatory control during sleep (1). CSA has multiple manifestations, including high altitude-induced periodic breathing, drug-induced central apnea, idiopathic CSA (ICSA), obesity hypoventilation syndrome (OHS), and Cheyne-Stokes breathing (CSB). Although the exact mechanism of induction of CSA may vary greatly among different types, an unstable ventilatory drive during sleep is a major underlying feature (2–5).

Central sleep apnea is rare in the general population, but is common in patients with heart failure, stroke, and atrial fibrillation (6, 7). In patients with HF, central sleep apnea is characterized by Cheyne-Stokes breathing, followed by apnea, mainly manifested by fatigue, insomnia, daytime sleepiness, and inattention (8). Not only that, central sleep apnea also has adverse effects on children. CSA occurs predominantly in children with underlying conditions (such as Chiari malformation), affecting less than 5% of healthy children (9, 10). At present, a series of treatment methods are used to treat CSA, mainly including phrenic nerve stimulation (11), adaptive servo ventilation (12), continuous positive airway pressure (13), etc., but there are still some defects. In recent years, the health risks exposed by central sleep apnea have attracted great attention from researchers, and there is more and more literature on this neighborhood. However, bibliometric research in this field is still a blank. Therefore, bibliometric analysis of publications, countries, institutions, journals, authors and keywords is needed to comprehensively summarize the overview of CSA.

Bibliometrics is the use of statistics to describe or show the relationship between published works, thus analysing published information (for example, books, journal articles, abstracts, keywords, citations, etc.). Bibliometrics can be used to measure the impact of research articles or to estimate the impact of selected research articles on future research (14). With the increasing importance of the amount of research literature and the impact of research, bibliometrics will continue to play an important role in assessing the impact of research.

This study aims to comprehensively summarize the current understanding and understanding of central sleep apnea, and conduct a bibliometric analysis of articles in this neighborhood, which can systematically evaluate the current research status and development trends in this field, so as to reveal the research hotspots and potential research gaps in this field.

## 2 Materials and methods

### 2.1 Data source and literature search strategy

We used the Web of Science (WOS) database for data collection. To improve the representativeness and accessibility of the data, we searched the Web of Science Core Collection (WoSCC). From January 1, 2004 to January 16, 2025, the search formula used was (TS = central sleep apnea), and a total of 4,726 articles were retrieved. To facilitate further analysis of the literature content, only articles and review articles were included in this study. The exclusion criteria were documents whose topics were unrelated to the research content of this study, withdrawn or repeatedly published. 1,687 documents were finally included, exported in the form of complete records, and saved as plain text files.

### 2.2 Software for bibliometric analysis

**Figure 1:**
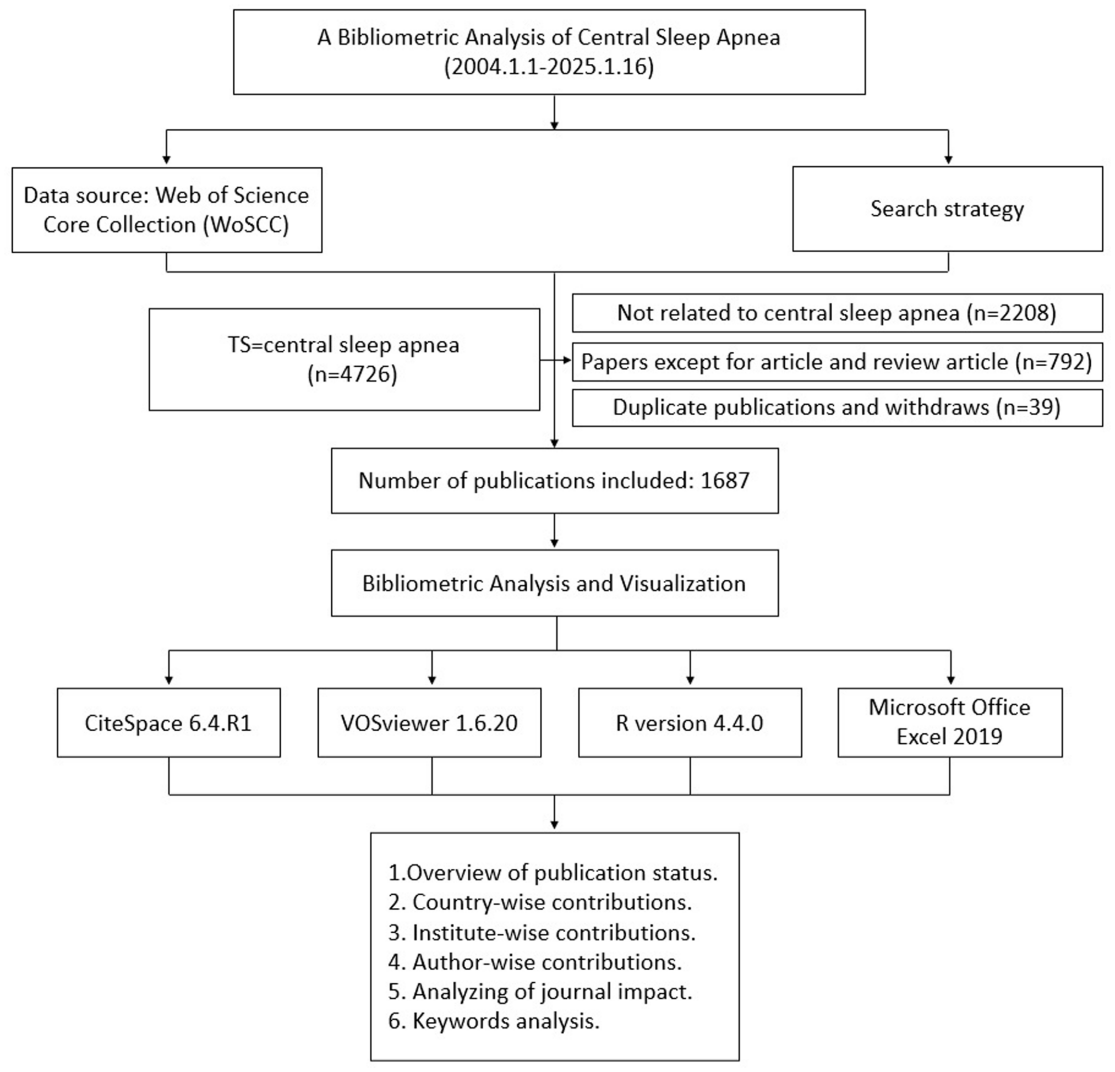
Flow chart of this study.

R (version 4.4.0), VOSviewer (version 1.6.20) and CiteSpace (version 6.4.R1) were used for data analytics and visualization (15, 16). VOSviewer was used for analysis and visualization of cooperation and keyword co-occurrence among countries, institutions and authors; CiteSpace was used to analyze journals and identify burst keywords, thus visually showing the basic knowledge and hot spots of the relationship between subject words and diseases, and predicting their research frontiers; The number of publications was analyzed using the linear growth function in Excel. Figure 1 shows the flow chart of the retrieval strategy and selection process in this study.

## 3 Result

### 3.1 Overview of publication status

**Figure 2:**
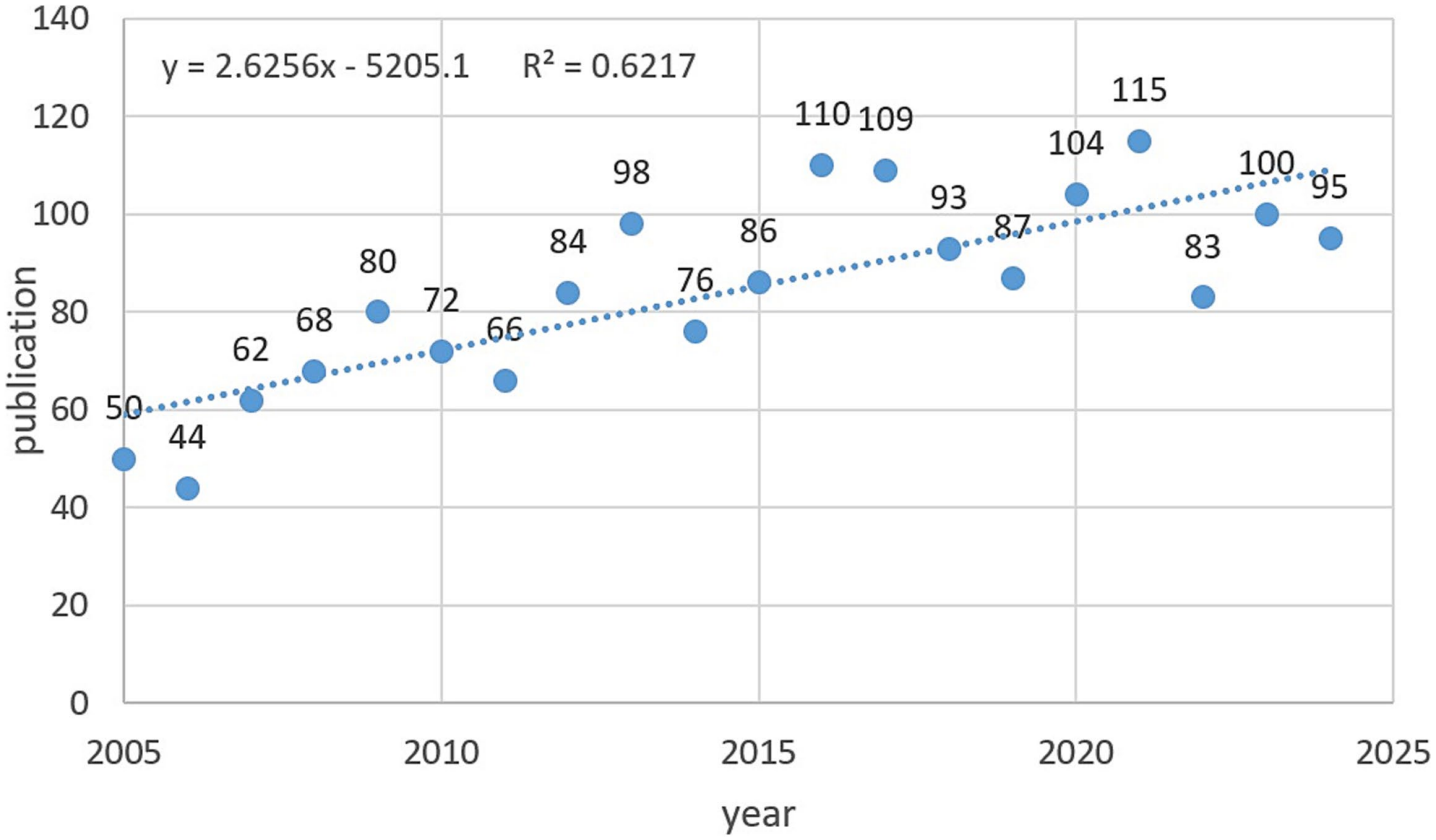
Published article trends.

A total of 1,687 articles on central sleep apnea were included in this study (Figure 1). The year and cumulative publication count related to them are shown in Figure 2. The number of publications is generally on the rise. There were fewer published papers from 2005 to 2015, and the number of publications each year was less than 100. However, from 2016 onwards, the number of annual publications gradually increased, reaching a peak in 2021 (115). The relationship between cumulative publications and the year of publication was evaluated using a linear growth function that matched the trend in the number of cumulative publications (R2 = 0.6217). The number of research publications in the field of central sleep apnea has shown a significant upward trend in the past 10 years, especially after 2016, reflecting a significant increase in research interest and activity in this field.

### 3.2 Analysis of national publications

**Figure 3:**
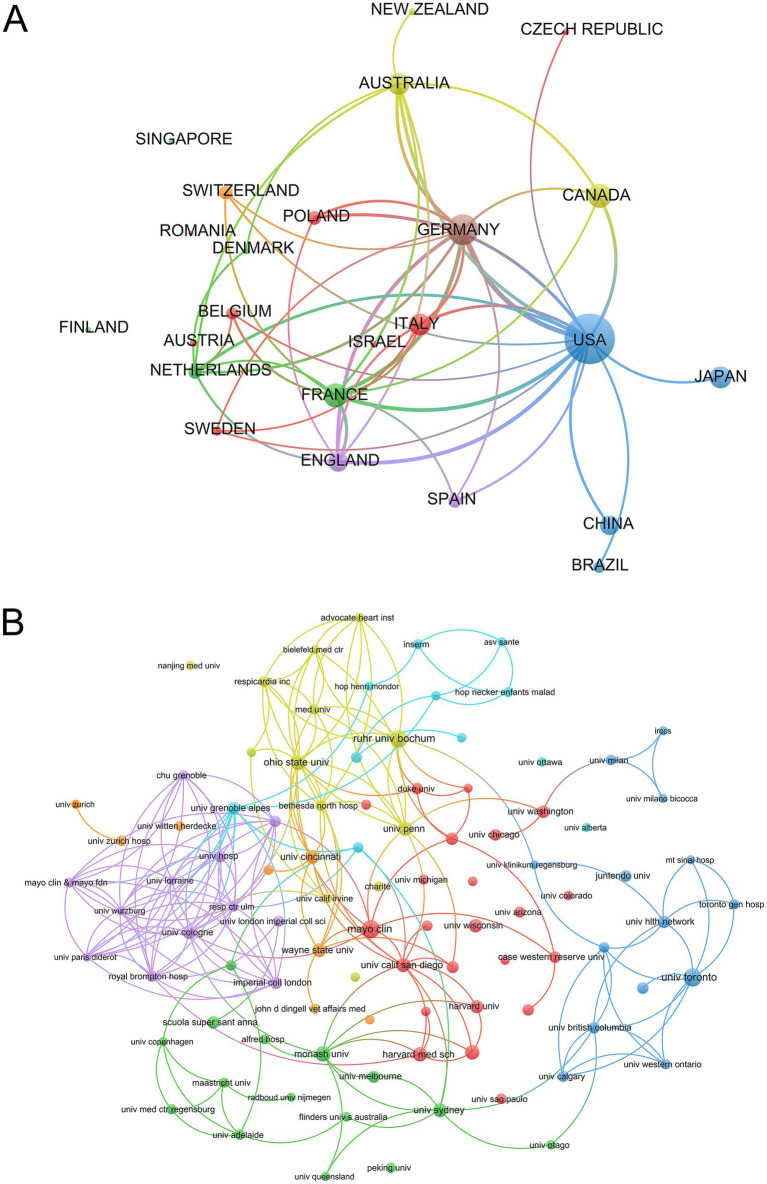
**(A)** Country cooperation of published articles. **(B)** Institutional publications.

Published literature in this field comes from a total of 42 countries/regions and is analyzed, as shown in Figure 3A. USA ranks first with 657 publications, followed by Germany (241), France (148), Canada (145), and the remaining countries/regions have less than 140 publications. The cooperation between countries/regions is analyzed based on VOSviewer software. Nodes represent countries/regions; node size indicates the number of posts, and the larger the node, the more posts; the color of the node indicates the clustering to which the country/region belongs; the lines between nodes indicate the existence of a cooperative relationship, and the thicker the lines, the closer the relationship, that is, USA and Germany cooperate most closely, followed by USA and France, England, Germany and France, indicating that these countries play a vital role in research in this field.

### 3.3 Analysis of institution publications

The institutional collaboration network view was used to identify institutions within a research area and their partnerships. To explore institutional contributions to central sleep apnea research, we analyzed partnerships between different institutions (Figure 3B), which were conducted at 75 institutions worldwide. The institutional Mayo clinic had the largest number of publications at 112, followed by the University of Toronto with 94. Harvard Medical School and Brigham and Women’s Hospital and the University of Toronto collaborated most closely with the University Health Network and The Hospital for Sick Children.

### 3.4 Analysis of journal impact

**Figure 4:**
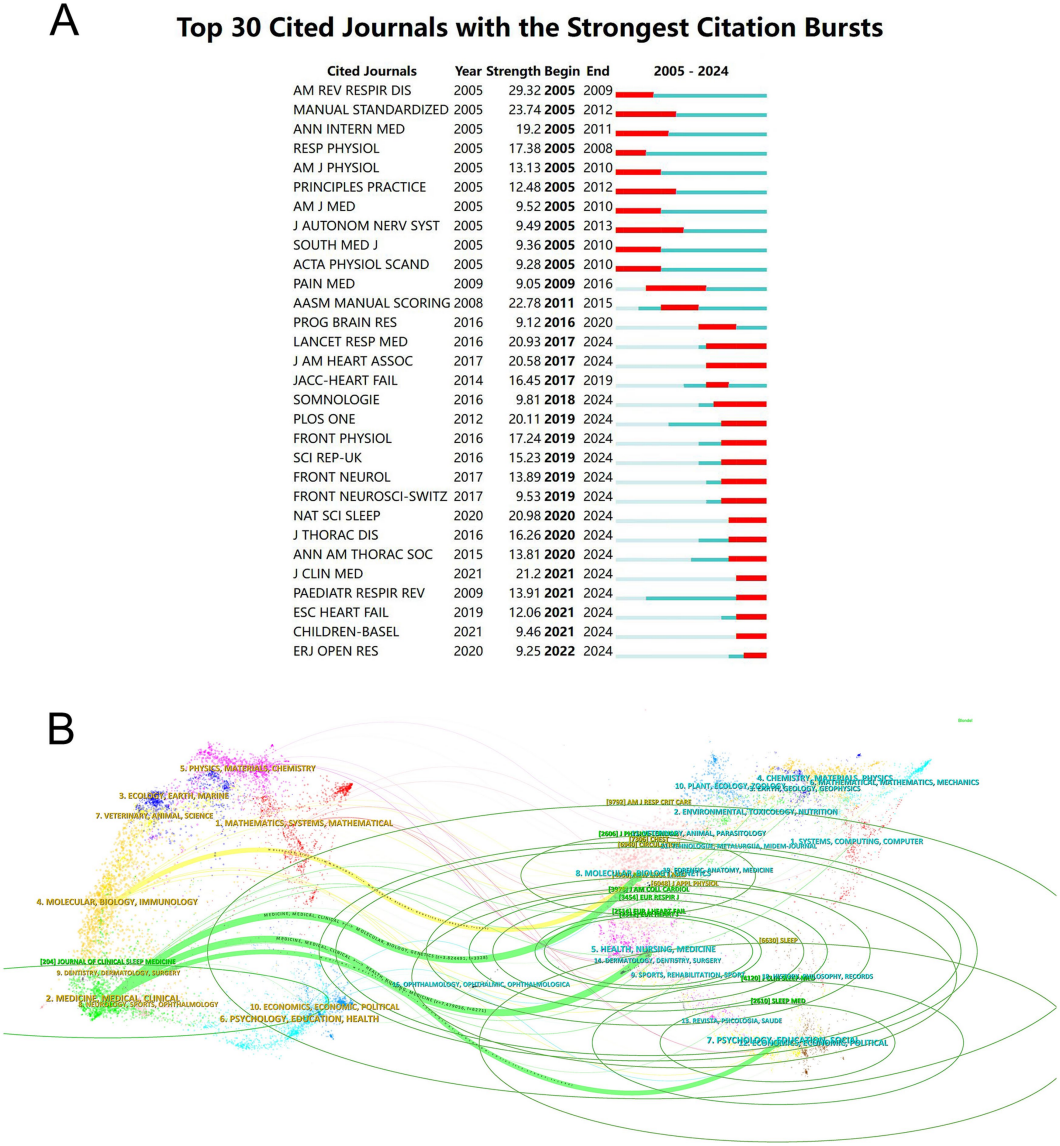
**(A)** Number of journal citations. **(B)** Dual-map overlay of journals.

A total of 903 journals were cited in the 1,687 articles on central sleep apnea included. The American Journal of Respiratory and Critical Care Medicine was the most cited journal (1,345), followed by CHEST (1,284), SLEEP (1,258), and The New England Journal of Medicine (1,028). The remaining journals all had less than 1,000 citations. We listed the 30 most cited journals, as shown in Figure 4A. The blue line represents the observation period 2004–2024, and the red line represents the outbreak time. The journal “American Review of Respiratory Disease” had the highest citation burst value (citation burst = 29.32) between 2005 and 2024.

The results of the superposition of the dual-map overlay of journals show the position of the research on this topic relative to the main research discipline. Each point on the graph represents a journal. The graph is divided into two parts. On the left is the citation graph and on the right is the cited graph. The curve in the middle is the citation curve, which completely shows the ins and outs of citation and provides an understanding of the interdisciplinary relationship in this field. The longer the longitudinal axis of the ellipse indicates that more papers are published in the journal, and the longer the horizontal axis indicates that the number of authors is more. The results of Figure 4B show that there are mainly 4 citation paths. The cited journals belong to the fields of “Medicine, Medical, Clinical” and “Molecular, Biology, Immunology.” The cited journals belong to the fields of “Molecular, Biology, Genetics,” “Health, Nursing, Medicine” and “Psychology, Education, Social.”

### 3.5 Author impact analysis

**Figure 5:**
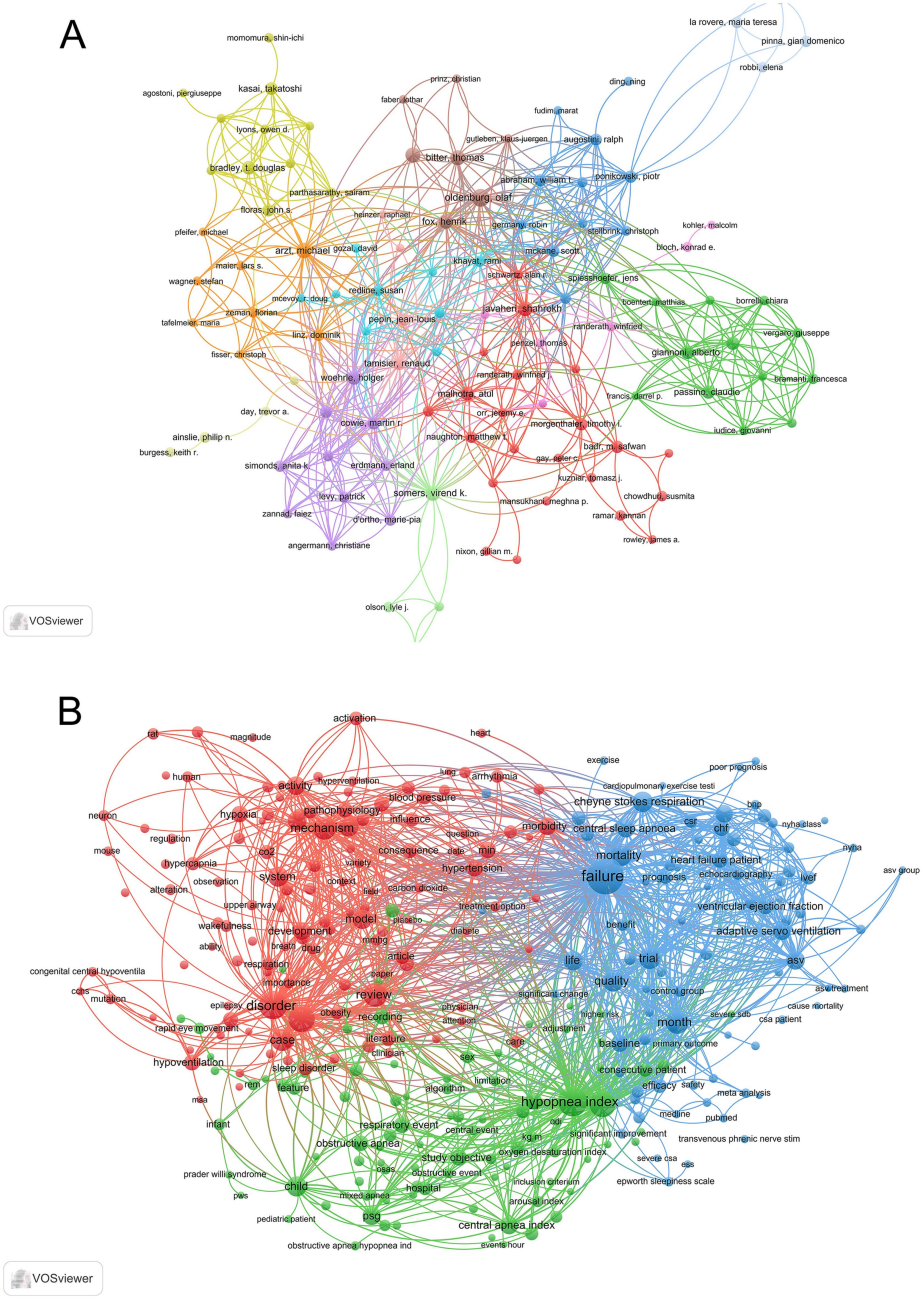
**(A)** Researcher collaboration. **(B)** Keyword co-occurrence analysis.

A total of 178 authors were involved in this field of research. Oldenburg and Olaf were the most published authors with 49 papers, followed by Arzt and Michael with 33 papers. Research author cooperation network view analysis is used to identify authors in a research field and their cooperation intensity, as shown in Figure 5A. The number of nodes indicates the number of authors, the size of nodes indicates the number of articles, and the thickness of connections indicates whether the cooperation relationship between authors is close. Oldenburg, Olaf, Fox, Henrik, Bitter, Thomas, Horstkotte, Dieter cooperated most closely, and Giannoni, Alberto, Emdin, Michele, Passino, Claudio also cooperated closely. Javaheri, Shahrokh, Germany, Robin, Augostini, Ralph, etc. constitute a high-yield author group, and clusters formed by multiple author groups can still be seen in the rest. This shows that author cooperation in the research field of central sleep apnea is relatively concentrated, and there is a certain connection between the research teams.

Keyword burst analysis Keywords are the condensation of the central idea of the paper and the soul of the paper. There are 734 key words in this studyof which 19 have appeared more than 100 times. Among them “central sleep apnea” (N = 731) is the most frequently used key word followed by “positive airway pressure” (N = 521) “heart failure” (N = 454) etc. The research status and hot spots in this field can be revealed by analyzing the key words. After co-occurrence analysis of keywords (Figure 5B) it was found that relevant research mainly focused on the following three directions: research on the relationship between central sleep apnea and heart failure involving keywords such as failure heart failure patients echocardiography ventricular ejection fraction and mortality; research on the mechanism of central sleep apnea involving keywords such as mechanism hypercapnia hypoventilation and upper airway; clinical research on central sleep apnea involving keywords such as hypopnea index central apnea index pediatric patients and inclusion criterium.

**Figure 6:**
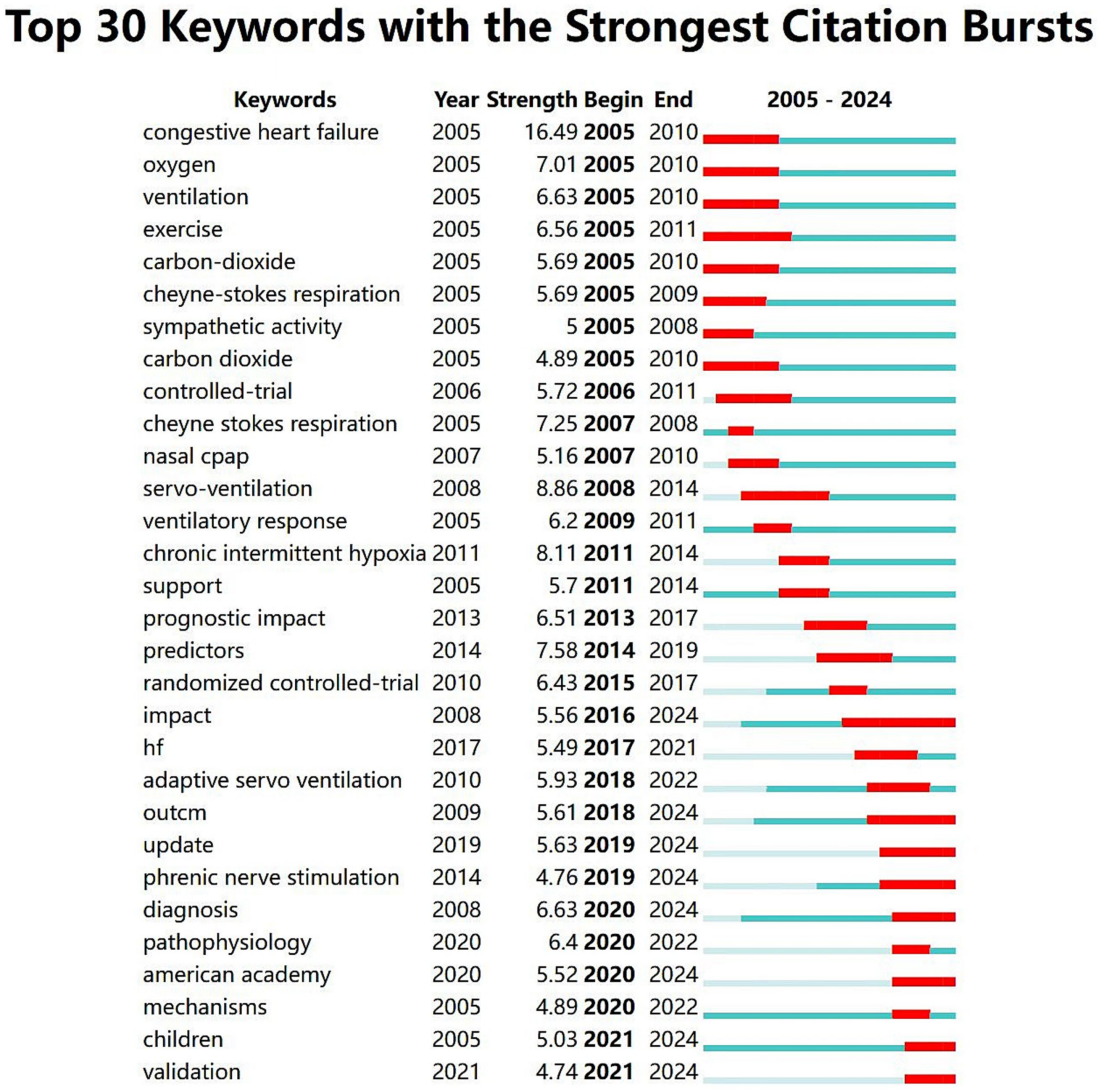
Top 30 keywords with the strongest citation bursts.

Keyword burst analysis can reflect the trend of research hotspots of central sleep apnea in a certain period of time with annual changes which is helpful to grasp the cutting-edge dynamics in this research field. As shown in Figure 6 the top 30 keywords with the strongest citation bursts in central sleep apnea are shown. The blue line represents the timeline that is the observation period from 2005 to 2024; the red part on the blue timeline indicates outbreak detection indicating the start year end year and burst duration. Key words Congestive heart failure (16.49) has the strongest emergent degree appearing in 2005; the emergent time of impact lasts the longest as long as 8 years indicating that the longest time to study the impact of central sleep apnea on human health; congestive heart failure is the earliest focus of researchers starting in 2005. Validation children mechanisms American academy pathophysiology diagnosis etc. have become research hotspots in recent years indicating that researchers have begun to pay attention to the mechanism of central sleep apnea on the human body, diagnosis, and research related to children.

## 4 Discussion

### 4.1 Basic information

The aim of this study was to explore the development trend and characteristics of central sleep apnea (CSA) -related research between 2004 and 2025 through bibliometric analysis. The results showed that CSA-related research has shown a rapid growth trend since 2004, especially in 2021. The annual number of articles reached 115, indicating that CSA-related issues are increasingly attracting researchers’ attention and interest. The main drivers of this growth trend include the increasing burden of disease, the increased risk of cardiovascular complications, diagnostic and treatment challenges, the growing demand for personalized medicine, and the improvement of public health awareness. These factors together prompt researchers to conduct more in-depth investigation and exploration of CSA-related issues, thus promoting the development and improvement of CSA research. In addition, this study also found that the fields and directions of CSA-related research are also expanding and diversifying, covering a variety of fields, such as clinical medicine, basic medicine, public health, statistics, etc. Researchers and experts in these fields jointly promote the progress and development of CSA research through cooperation and exchanges.

A total of 1,687 academic papers from 42 countries/regions were included in this study, and the geographical distribution and international cooperation patterns of global CSA research were analyzed. The results showed that the United States and Germany dominated the number of CSA research papers published, showing the scientific research strength of these two countries in this field. Further analysis found that the United States-centered international cooperation occupied the main position in the international cooperation network, reflecting the central role and international influence of the United States in the field of CSA research. This leading position may be attributed to the robust economic strength of the United States and the continuous high level of medical and health investment, which provides a solid material guarantee and talent base for basic research, clinical research and research and development of new technologies in CSA. High-level scientific research facilities, sufficient scientific research funds and relatively perfect research systems all provide favorable conditions for the United States to maintain its leading position in the field of CSA research. However, it is worth noting that other countries are also actively participating in CSA research, and the increasing frequency of international cooperation has also promoted the sharing and development of knowledge in this field.

Of the 75 institutions included in this study, 18 of the top 30 institutions are located in the United States. This proportion is similar to the distribution of the number of published papers by country/region, which once again confirms the leading position of the United States in the field of CSA research. Although Germany ranks second in the number of published papers, only 3 universities have entered the top 30, indicating that Germany’s research power is relatively concentrated in a few top institutions. France ranks third in the number of publications, and only 3 institutions have entered the top 30, showing that France also has a relatively concentrated distribution of institutions in CSA research. In contrast, Canada’s Univ Toronto ranked second with 94 papers, becoming an important contributor to global CSA research. This phenomenon shows that although economy and resources are important factors limiting research output, various institutions are actively seeking international cooperation to improve their scientific and technological competitiveness. Through international cooperation, institutions in various countries can share resources, technologies, and talents, thereby improving research quality and output. In addition, these phenomena also reflect the trend of global scientific research cooperation. With the complexity of scientific research and the increasing demand for interdisciplinary, it is difficult for a single institution or country to independently complete high-level research. International cooperation not only helps to break through geographical and resource constraints, but also promotes the sharing of knowledge and the generation of innovation. Therefore, the active cooperation of institutions in various countries in the field of CSA research not only improves their respective scientific research capabilities, but also promotes the development of the entire field.

Peer-reviewed journals play a vital role in academic publishing, and they play a key role in ensuring the quality of research and academic norms. In the field of CSA research, published articles in core journals often represent the latest progress and important research results in this field, providing valuable reference and reference for researchers. When selecting journals to publish papers, researchers usually refer to the number and influence of papers published by the journal in the field of CSA to judge the suitability and scope of influence of their journals. The results of this study show that the American Journal of Respiratory and Critical Care (AM J RESP CRIT CARE) is the journal with the largest number of CSA-related research papers published, which indicates that the journal has significant influence and authority in the field of CSA research and is one of the important choices for researchers to publish CSA-related research results. In addition, we also need to pay attention to the contribution of China in CSA. As the largest developing country in the world, China has played an important role in the research and development of this field. We propose to encourage global researchers to establish cooperative relationships and participate in international multi-center research projects. Promote the open sharing of CSA research data and accelerate scientific research progress. Use emerging technologies such as artificial intelligence, big data and genomics to promote CSA research.

### 4.2 Research focus and hotspots

The keywords of research papers are highly refined results of the authors’ academic achievements, guiding the research direction, academic topics, publishing framework and core papers. These keywords not only reflect the authors’ research interests and expertise, but also predict future research topics and hotspots, which play a key role for researchers to explore changes and emerging trends in the discipline. In this study, the keywords were analyzed using VOSviewer software. We obtained the following 3 research areas and corresponding research hotspots:

Mechanisms of interaction between CSA and heart failure: The association between CSA and congestive heart failure (CHF) has been widely recognized. CHF may predispose individuals to CSA, which in turn may exacerbate CHF progression (17). In clinical practice, CSA in congestive heart failure usually occurs in conjunction with decreased exercise capacity, reduced ejection fraction, increased left ventricular volume, and elevated pulmonary capillary wedge pressure, collectively suggesting that CSA is more likely to occur in later stages of congestive heart failure (18). At the same time, many risk factors for CSA in patients with CHF have been identified, including male gender, higher New York Heart Association (NYHA) functional class, lower ejection fraction, conscious hypocapnia (arterial partial pressure of carbon dioxide [PaCO_2_] < 38 mm Hg), higher prevalence of atrial fibrillation, higher B-type natriuretic peptide levels, and frequent nocturnal ventricular arrhythmias (19–22). Numerous studies have shown that increased respiratory control responses to changes in PaCO_2_ above and below the apnea threshold are central to CSA pathogenesis in CHF (23–25). Future research can further explore the bidirectional mechanism between CSA and CHF, especially the role of respiratory control response, hypocapnia and hemodynamic changes in it. Use omics technology to accelerate the search for biomarkers related to CSA and CHF to identify high-risk patients early and guide personalized treatment. Strengthen multidisciplinary collaboration in research, including cooperation in respiratory, cardiovascular, neuroscience and engineering fields.

Mechanisms of CSA: The basic mechanism of CSA may be due to hypoventilation or hyperventilation. Revocation of the awake respiratory drive results in reduced ventilatory motor output, which may not matter in healthy individuals, but can lead to hypoventilation or even apnea or thoracic disease in patients with insufficient ventilatory reserve (eg. patients with neuromuscular disease) (26). CSA comes in several forms. If altitude is high enough, most people who rise to high altitude will develop periodic breathing (27). This is an unstable form of ventilation produced by environmental hypoxia (low air pressure). Hypoxia at high altitude leads to hyperventilation and hypocapnia. As a result, ventilation fluctuates between apnea and shortness of breath. For this, there must be an adequate hypoxic ventilatory response to drive hyperventilation, leading to hypocapnia (28). Idiopathic CSA is a relatively uncommon condition, usually with an elevated hypercapnic ventilatory response. This breathing disorder occurs predominantly during NREM sleep because chemosensitivity decreases during REM sleep and is not sufficient to drive cyclic breathing. These central apneas can be eliminated by CO_2_ inhalation (29). The third form of CSA, called Cheyne-Stokes respiration, usually occurs in patients with congestive heart failure and is the result of a combination of high CO_2_ responsiveness, hypocapnia due to high filling pressure, and long circulation time (30–33). Future research may focus on the chemosensitivity of the respiratory center, neuroregulatory mechanisms, and the molecular basis of ventilatory instability. For different types of CSA, studying their unique pathophysiological mechanisms and personalized treatment plans is essential. Use artificial intelligence, big data and wearable technology to develop intelligent diagnostic and therapeutic tools to achieve early screening and dynamic monitoring of CSA.

Clinical studies related to CSA: Patients with CSA have a wide range of potential diagnoses, including hypoxic–ischemic encephalopathy, Ondine syndrome, Prader-Willi syndrome, primary central apnea, brain tumors, and Down syndrome (34). Studies have shown that patients with central apnea exhibit higher central apnea-hypopnea indices (cAHI), and in children with central apnea and adenotonsillar hypertrophy, adenotonsillectomy has been shown to improve central apnea (35). In addition, CSA has a large impact on HF. Without treatment, patients with CSA have twice as much mortality and morbidity as patients with HF without sleep apnea, and symptoms overlap with HF (36, 37). Treatment options for CSA are few and focus primarily on adaptive servoventilation, which has no effect on morbidity and mortality and limited impact on quality of life in randomized controlled trials (38, 39). Recent studies have also found that phrenic nerve stimulation is an effective treatment for CSA, as measured by improvements in overall AHI, central apnea index (CAI), and oxygen desaturation index (ODI) (40). Future studies can explore the common pathophysiological mechanisms of CSA in these diseases, especially the role of neuroregulation and respiratory center dysfunction. To evaluate the long-term efficacy and safety of adenotonsillectomy and explore other potential treatment methods. In-depth study of the impact of CSA on mortality, morbidity and quality of life in patients with HF, and explore the interaction mechanism between CSA and HF. The interaction mechanism between CSA and HF is complex and involves multiple physiological systems. It is recommended to strengthen multidisciplinary collaboration and integrate research resources in respiratory, cardiovascular and neuroscience fields.

At present, the study still has some limitations, which need to be considered when interpreting and applying the results. First, the WoSCC database is a globally recognized database of scientific publications, which has the characteristics of high quality and wide coverage. In order to guarantee the accuracy and reliability of the analysis, the data analytics of this study is only based on the WoSCC core collection. Nevertheless, this choice may ignore some high-quality studies that are not included in the WoSCC core collection, but this does not affect the overall trend and main conclusions of the results. Secondly, this study uses tools such as CiteSpace and VOSviewer for keyword frequency statistics, co-occurrence analysis and cluster analysis, which are of great value in revealing research hotspots and trends. However, these tools cannot completely replace systematic literature search and manual screening. Researchers still need to combine traditional literature search methods in practice to ensure that important research results are not missed. Newly published high-quality research may not be introduced into the analysis in time due to some factors. For example, newly published articles may not have been widely cited, or the update lag of databases and other reasons, resulting in these studies have not been included in the current analysis. Therefore, timely updates and supplements in follow-up studies are needed to ensure the timeliness and comprehensiveness of the results. Overall, despite the above limitations, our study still lays a solid foundation for researchers to understand the research topics, hot spots and development trends of CSA. Through systematic bibliometric analysis, we reveal the current research status and future directions in this field, providing valuable references and guidance for related research. In future research, we will continue to pay attention to the latest progress in this field and timely update the analysis results to further improve and deepen the understanding of CSA.

## 5 Conclusion

Through bibliometrics and visual analysis methods, this study summarizes and analyzes the relevant literature in the field of CSA since 2004, summarizes the research status in this field, and focuses on its research hotspots and development trends, which is conducive to researchers to dig deep into the current research results and promote the development of related research in this field. This bibliometric analysis underscores the rapid growth of CSA research, driven by its cardiovascular implications and diagnostic challenges. Key findings include the dominance of U.S. and German institutions, robust author collaborations, and evolving research foci on CSA-heart failure interactions, ventilatory instability mechanisms, and therapeutic innovations. Emerging keywords (e.g., children, pathophysiology) signal shifting priorities toward pediatric populations and molecular mechanisms. Despite advancements, gaps persist in understanding bidirectional CSA-CHF pathways, long-term treatment efficacy, and biomarker discovery. Future studies should leverage omics, AI, and wearable technologies to enhance early diagnosis and dynamic monitoring. Strengthening global collaborations and integrating multidisciplinary expertise (e.g., cardiology, neuroscience) will accelerate breakthroughs. While limited by database coverage, this study provides a comprehensive roadmap for addressing CSA’s clinical and mechanistic complexities, ultimately improving patient outcomes through targeted, evidence-based interventions.
